# 2-year results of middle-aged patients with two-compartment cartilage lesions in one knee treated with two patient specific metal implants

**DOI:** 10.1186/s40634-023-00648-2

**Published:** 2023-09-14

**Authors:** Daniel Aaron den Toom, Markus Rieke, Allaeldin Elbadawi, Clemens Kösters, Maria-Josef-Hospital Greven

**Affiliations:** Maria-Josef Hospital Greven, Greven, Germany

**Keywords:** Patient specific implant, Knee preservation, Focal cartilage lesion, Middle-aged patient, VAS for pain, KOOS, Arthroplasty, Best-ager, Functional outcome, Cartilage repair

## Abstract

**Purpose:**

Focal chondral lesions of the femur are currently treated with biological repair or arthroplasty. However, some patients are not suitable for either one due to lesion size, age, or prior biological treatment attempts. While singular patient-specific focal mini metal implants already showed good results, the outcomes of bicompartmental implantation of these implants have not been discussed in the literature yet. This study aims to evaluate clinical outcomes of patients who underwent bicompartmental implantation of two patient-specific implants.

**Methods:**

This prospective, non-randomized, non-comparative pilot study evaluates results up to two years after bicompartmental implantation of two implants (Episealer Implant, Episurf, Stockholm, Sweden).

A damage report is compiled using a special MRI program and patient specific implants are manufactured, including 3D-printed surgical instruments to provide exact placement of the implant.

The patients were assessed repeatedly using the Knee Injury and Osteoarthritis Outcome Score (KOOS) and Visual Analogue Scale (VAS) for pain during the follow-up.

**Results:**

The scores were evaluated three, 12, and 24 months after surgery and showed good results. The median in both scores improved from 37.7 for the KOOS5 preoperatively to 69.1 after 24 months and from 69 for the VAS for pain preoperatively to 9 after 24 months.

**Conclusion:**

Overall, for the small study group presented, the early results are promising. With noticeable improvement in KOOS and VAS for pain after two years, patient specific implants appear to become relevant in future standardized treatment of femoral chondral lesions. Especially with bicompartmental implantation, full arthroplasty can be delayed even further.

**Level of Evidence:**

IV

**Supplementary Information:**

The online version contains supplementary material available at 10.1186/s40634-023-00648-2.

## Introduction

Focal chondral lesions in the knee can heavily affect a patient’s mobility due to severe pain and restrictions in daily life [22]. Moreover, the lesions increase the risk for osteoarthritis, and thus proper initial treatment is required [10].

The current options include bone marrow stimulating techniques, such as microfracturing or autologous matrix-induced chondrogenesis (AMIC), autologous chondrocyte implantation (ACI), or osteochondral allografts (OCA) [13]. All procedures achieve good results, especially in younger patients. With increasing age, however, results become less promising [3, 15]. Viable biological options for the “best-agers”, patients between around 40 – 60 years, or revision cases are not available. Nevertheless, oftentimes a partial or full arthroplasty is not optimal for these patients either, since the rates for early implant loosening are high [5, 7]. In 2023, Perdisa et al. found that revision rates in total knee arthroplasty were twice as high for patients younger than 65 years compared to older patients [19].

This creates a treatment gap, with patients being too young for an arthroplasty but too old for biological chondral repair. Mini metal implants have been developed for these patients, but they showed high revision rates of 23% after seven years and subsequently underwent arthroplasty [12].

Exact and stable positioning of the implant seems to be required to avoid an uneven pressure distribution on the opposing cartilage.

A new patient-specific mini metal implant has been developed to allow for exact implant positioning based on the patient’s MRI- scan with 3D-printed operation material.

The results over the first five years appear to be promising. Al-Bayati et al. examined a series of 10 cases, aged 30 – 65, with a minimum follow-up of five years. All patients showed significant improvement of their VAS for pain (*p* ≤ 0.001) and KOOS sub scores for pain (*p* = 0.01), activities of daily life (*p* = 0.003), sport and recreation (*p* = 0.024) and quality of life (*p* = 0.003) [1]. In certain cases, however, coverage of two chondral lesions is required. Thus, this study introduces the implant, including indications and surgical technique. Considering the good results of singular patient-specific implants over the first five years, this study is the first to investigate the outcome of bicompartmental implantation and presents the first short-term results of patients treated with two patient-specific implants in one knee.

## Materials and methods

### Implant

**Fig. 1:**
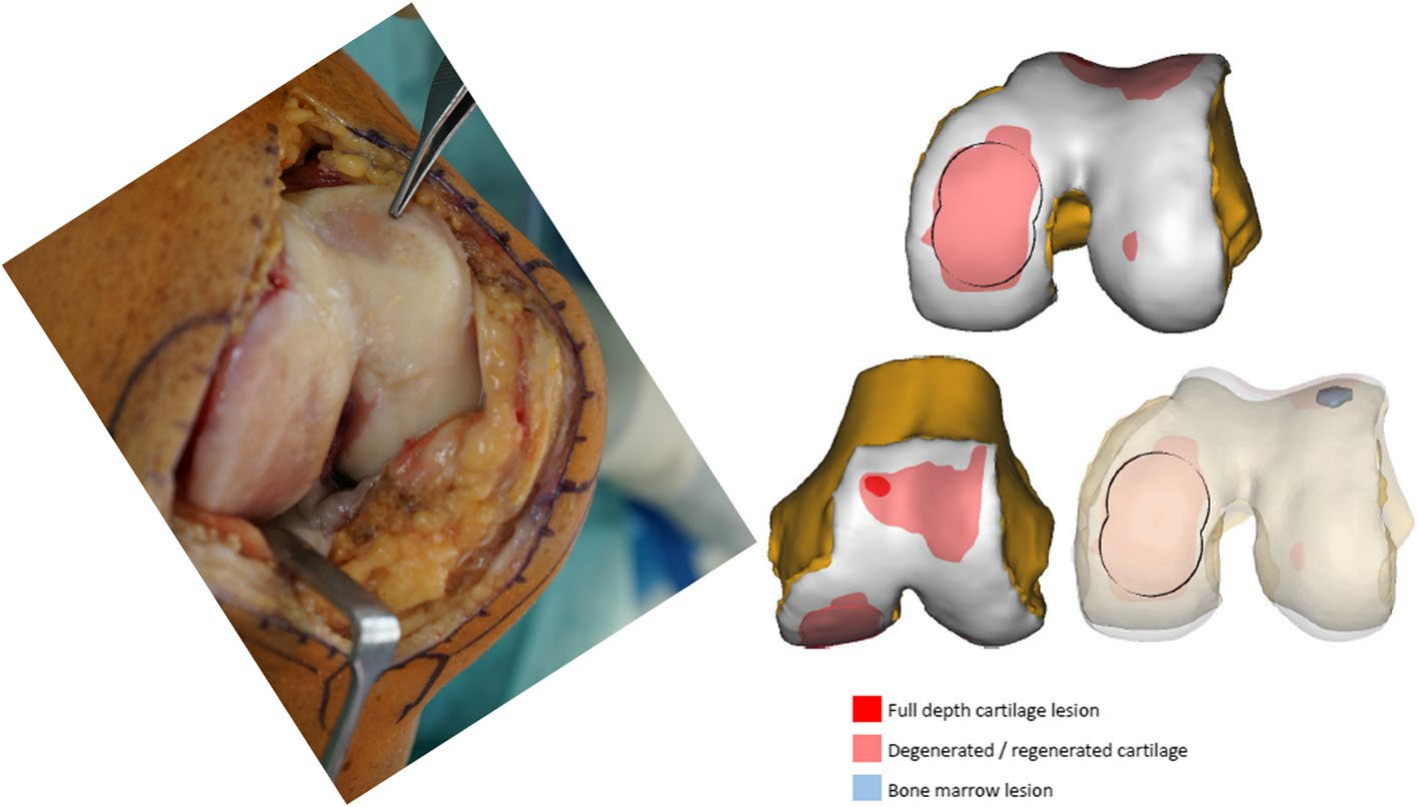
Damage marking report compared to the intraoperative view on the patient’s knee

**Fig. 2:**
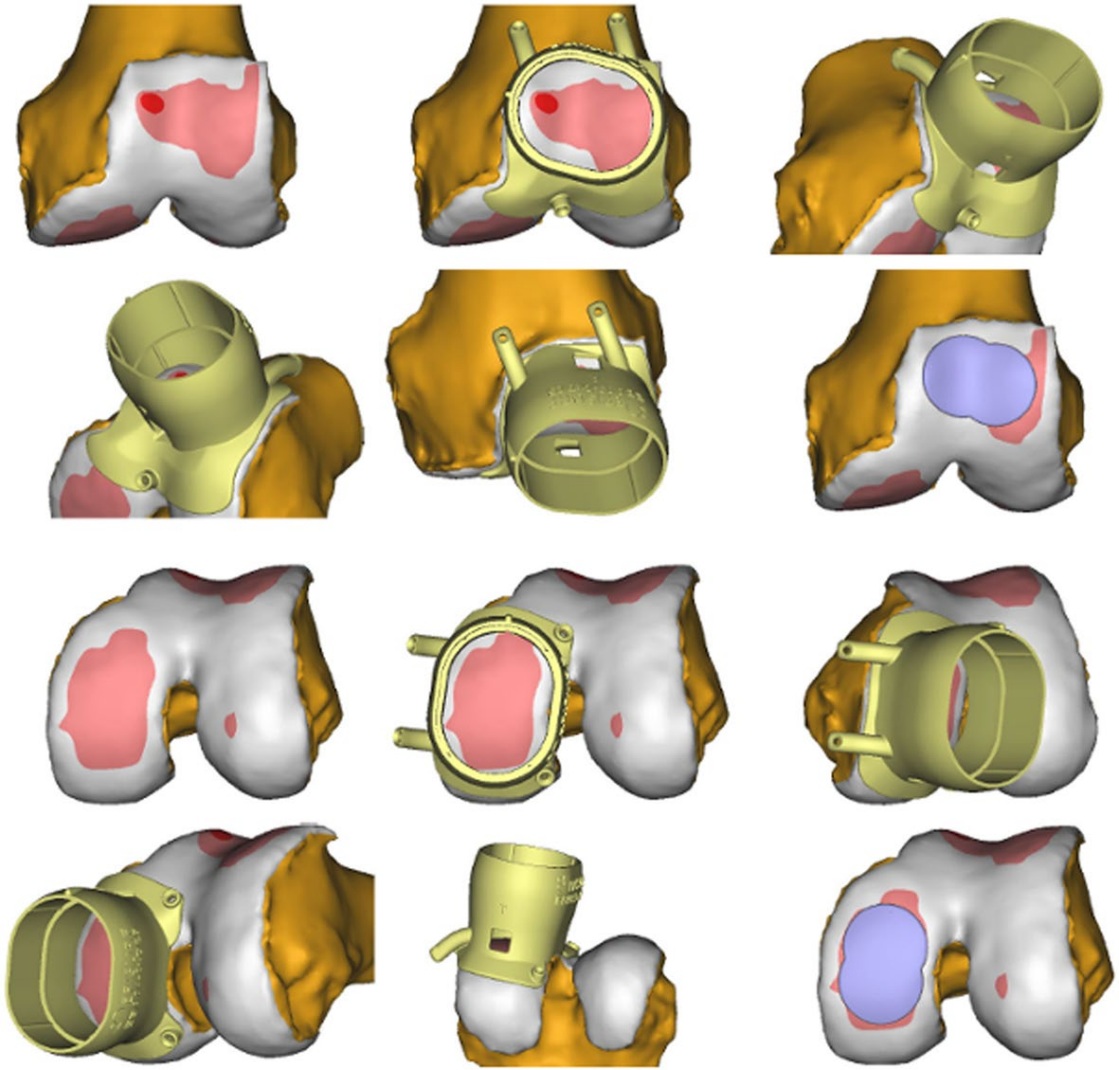
Damage marking report showing the exact placement of the 3D-printed “Epiguide” for implant positioning

**Fig. 3:**
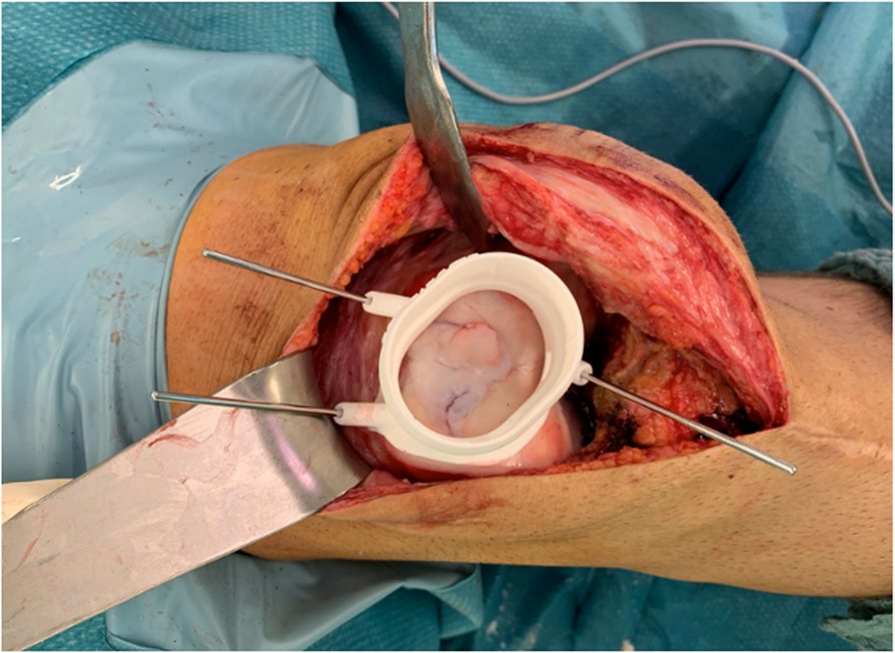
Intraoperative placing of the drill guide

The mini-metal implant (Episealer Implant, Episurf, Stockholm, Sweden) consists of a cobalt chrome alloy with a titan and hydroxyapatite coating to ensure faster and permanent adherence to bone. It is planned based on a damage marking report compiled from a special MRI-program according to Episurf's proprietary software for segmentation and 3D visualisation showing the lesions in a virtual 3D-model (Figs. 1 and 2). The data are then reviewed by the surgeon, and after giving permission, the final implant is produced in three to five weeks. Additional patient-specific 3D-printed surgical instruments provide exact placement of the implant and adjustment of the drilling depth in 0.2 mm steps (Figs. 2 and 3).

**Fig. 4:**
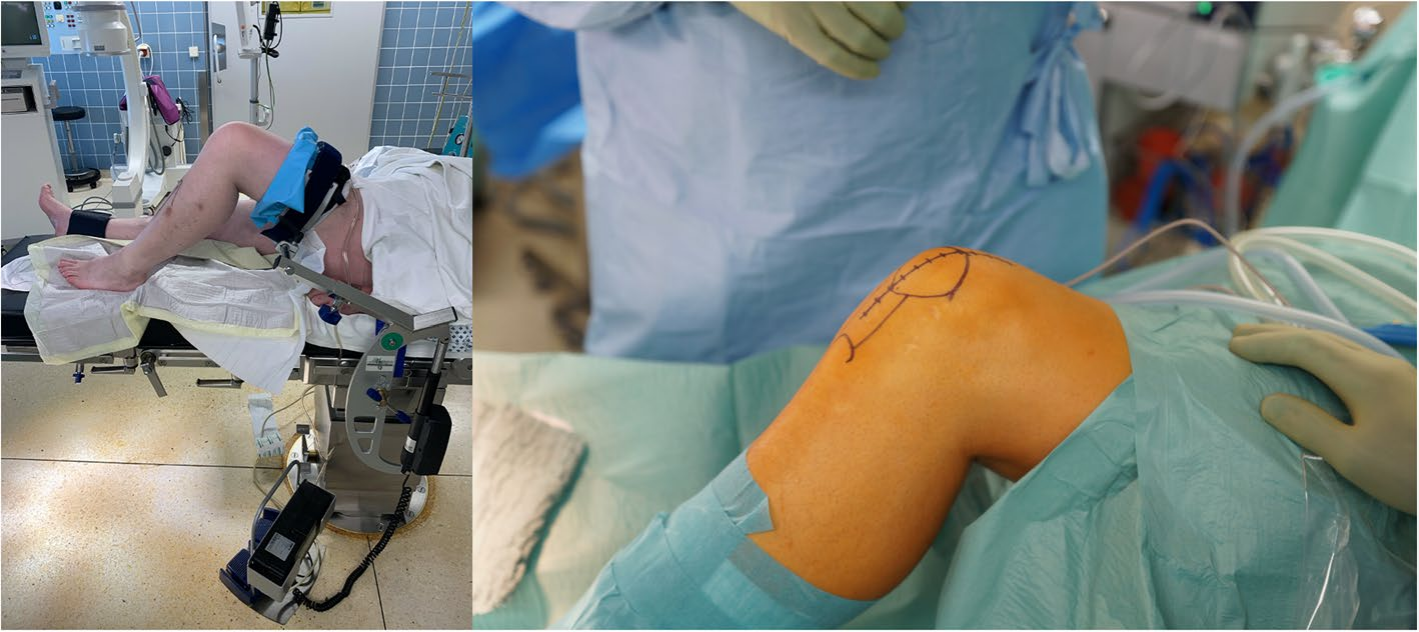
Intraoperative setup with a non-tightened torniquet

Depending on the affected area, the surgical approach is performed similarly to a total knee replacement. An adjustable electric limb positioner and a non-tightened torniquet can be used. To provide exact placement of the 3D-printed tools intraoperatively, an anteromedial or lateral approach corresponding to the affected femoral condyle is recommended (Fig. 4).

After the osteochondral lesion is fully visible, the drill guide is aligned to the bone following the 3D-image. The guide is then fixed with k-wires and a first depth-drilling is performed. Afterwards a patient-specific dummy of the implant is inserted, and the insertion depth is verified with the backside of a k-wire. Ideally, the implant is placed around 0.5 to 1.0 mm below the surrounding cartilage level. The depth can be further adjusted with a drilling socket in 0.2 mm steps to allow for exact implant positioning.

**Fig. 5:**
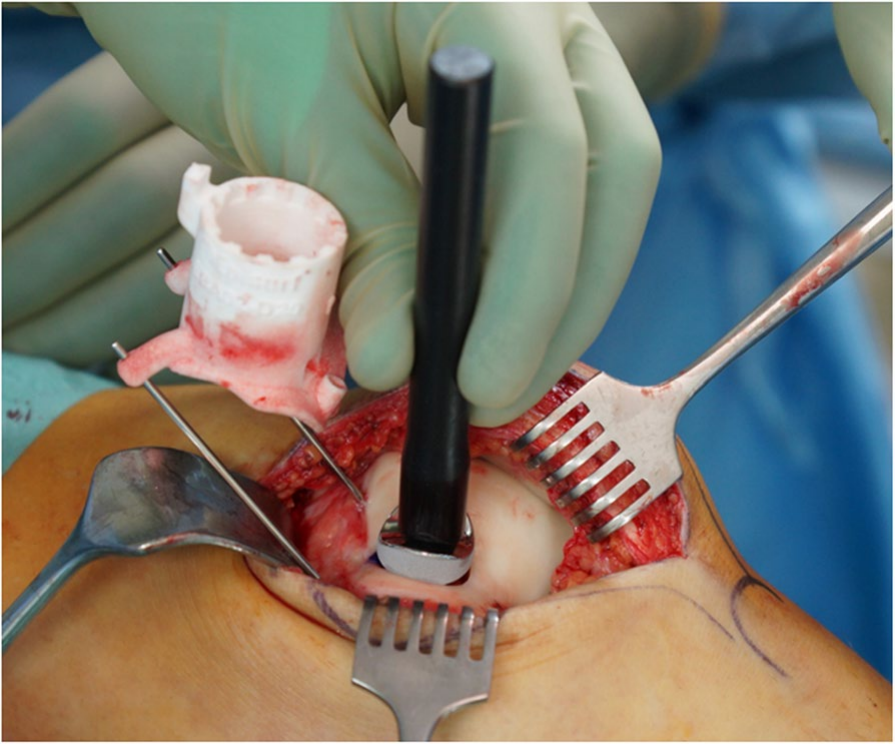
The implant is tapped down with a mandrel

**Fig. 6:**
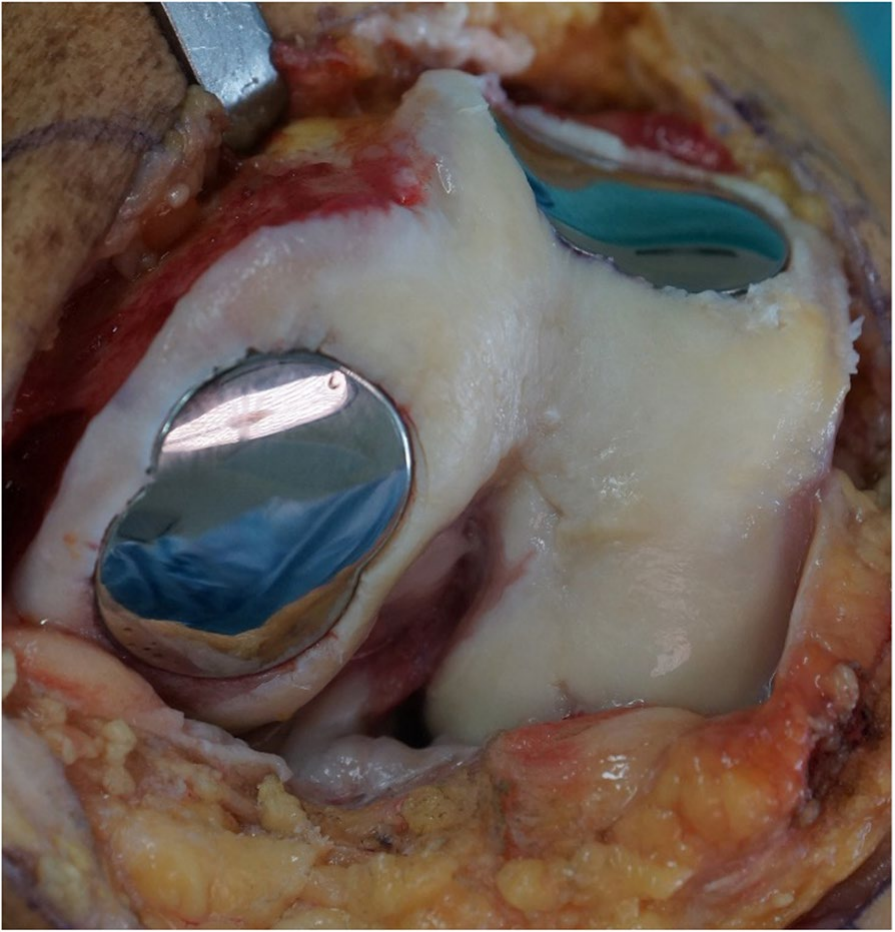
Two patient-specific implants in one knee

**Fig. 7:**
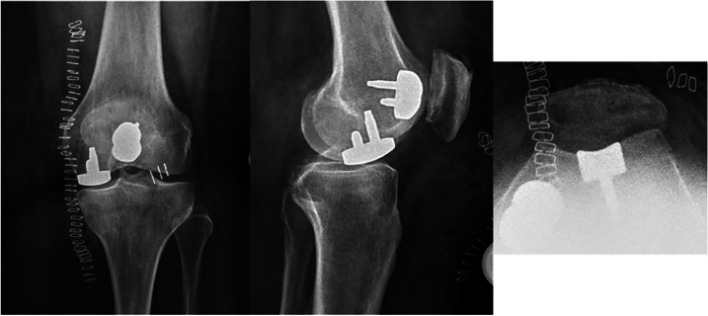
Postoperative x-ray after implantation of two patient-specific implants

The implant is then tapped down with a patient-specific mandrel in a press-fit fixation until it is fully seated (Figs. 5, 6 and 7).

### Indications and contraindications

Isolated focal chondral lesions of up to 7.3 cm^2^ (as recommended by Episurf, Stockholm, Sweden) of the femoral condyle or trochlea can be treated with the patient-specific mini metal implants. In younger and middle-aged patients, with respect to biological age, biological chondral repair is still considered the treatment of choice. The implant should be considered especially in revision cases with failed biological chondral repair or in older patients.

Contraindications include damage of the opposing tibial or patellar cartilage (ICRS >2), axial deviation of more than 5°, infections, rheumatic joint disease, immunosuppression, and severe osteoporosis.

### Concept of the study

Patient data for this prospective, non-comparative single-center pilot study was collected from 2019 to 2021. The minimum follow-up was 12 months (*n* = 23), and the maximum follow-up was 24 months (*n* = 11). Patients undergoing surgery were recruited to participate in the study. Informed consent was obtained for each patient. Demographic data, VAS (Visual Analogue Scale) and KOOS (Knee Injury and Osteoarthritis Outcome Score) were collected postoperatively from patients at three, 12, and 24 months. One experienced senior surgeon performed the surgery on all patients. At the time of surgery, all patients suffered from a ICRS (International Cartilage Research Society) grade of four.

Postoperatively, patients were partially weight bearing (20 kg) for ten days and fully weight bearing after. Patients were dismissed from the hospital once they achieved a range of motion (ROM) of at least 0/0/90° and were able to climb stairs.

Patient data were collected in regular postoperative follow-up examinations without any further procedures outside of the standards conducted. Therefore, ethical approval was not requested.

### Inclusion criteria

Patients between 39 and 65 years who met the above-mentioned criteria for indications and contraindications were included in this study.

### Statistics

Descriptive statistics were calculated, and graphs were created using Microsoft Excel (Microsoft, Redmond Washington USA). Further statistical tests were not conducted due to the small patient collective and subsequent lack of statistical power to detect effects.

## Results

### Patient collective

Data of 23 patients were prospectively collected three and 12 months postoperatively Table 1. Eleven of 23 patients were available for follow-up at 24 months. All patients underwent treatment with two patient-specific implants of the medial and lateral femoral condyle (*n* = 2), the lateral femoral condyle and the trochlea (*n* = 2), the medial femoral condyle and the trochlea (*n* = 18), or a double implant of the trochlea (*n* = 1). Demographic data, the Knee Injury and Osteoarthritis Outcome Score (KOOS) and the Visual Analogue Scale for pain (VAS) were gathered.

**Table 1:** Preoperative characteristics of the cohort (*n* = 23)

	Male	Female
Number	8	15
Mean age	54	52
Mean BMI	28	27.3
N with lesion of medial condyle	5	15
N with lesion of lateral condyle	3	1
N with lesion of trochlea	8	14

Fifteen female and eight male patients, with a mean age of 53 years (SD = 7.3), and mean BMI of 28 (SD = 3.7), participated in this study. One patient was younger than 40 years (at 39 years-old), and five patients were older than 60 years.

**Fig. 8:**
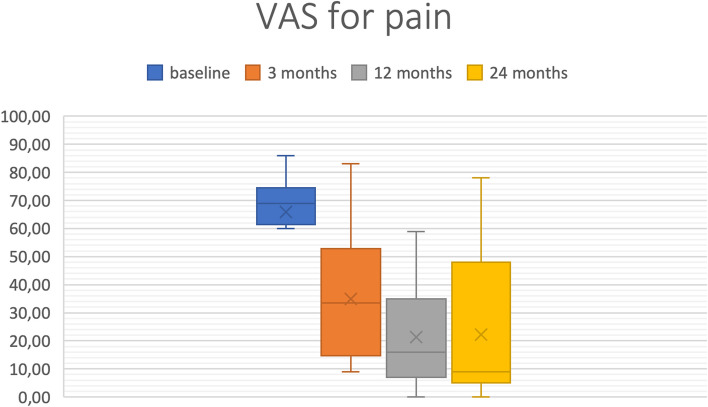
Boxplot: Visual Analogue Scale (VAS) for Pain average n = 23 (*n* = 11 at 24 months)

The VAS for pain showed a recognizable increase Table 2. 75% of the patients initially reported a VAS for pain higher than 61.5 (median = 69). After three months, 75% of the patients presented with a VAS for pain lower than 52.8 (median = 33.5), after 12 months lower than 35 (median = 16), and after 24 months lower than 48 (median = 9) (Fig. 8).

**Table 2:** Statistical results for KOOS5 and VAS for pain including mean, median, range, and standard deviation (SD), as well as first and third quartile (*n* = 23 after one year, *n* = 11 after two years). The number in the columns headings refers to the number of months postoperatively

	**VAS preOP**	**VAS3**	**VAS12**	**VAS24**	**KOOS5 preOP**	**KOOS5 3 months**	**KOOS5 12 months**	**KOOS5 24 months**
Min	35	9	0	0	17.4	10.5	27.4	26.8
Max	86	83	59	78	63.8	79.5	93.4	97.9
Mean	65.8	34.9	21.3	22.3	38.5	53.2	66.7	69.4
Standard deviation	13.7	22	18.4	25.6	13.1	19	19.5	20.8
1st quartile (exclusive)	61.5	14.8	7	5	28.3	43.3	53.6	57.6
Median	69	33.5	16	9	37.7	51.7	64.7	69.1
3rd quartile (exclusive)	74.5	52.8	35	48	45.3	72.8	84.3	88

*VAS* Visual Analogue Scale for Pain

*KOOS* Knee Injury and Osteoarthritis Outcome Score; KOOS5 represents the mean of all five subcategories

**Fig. 9:**
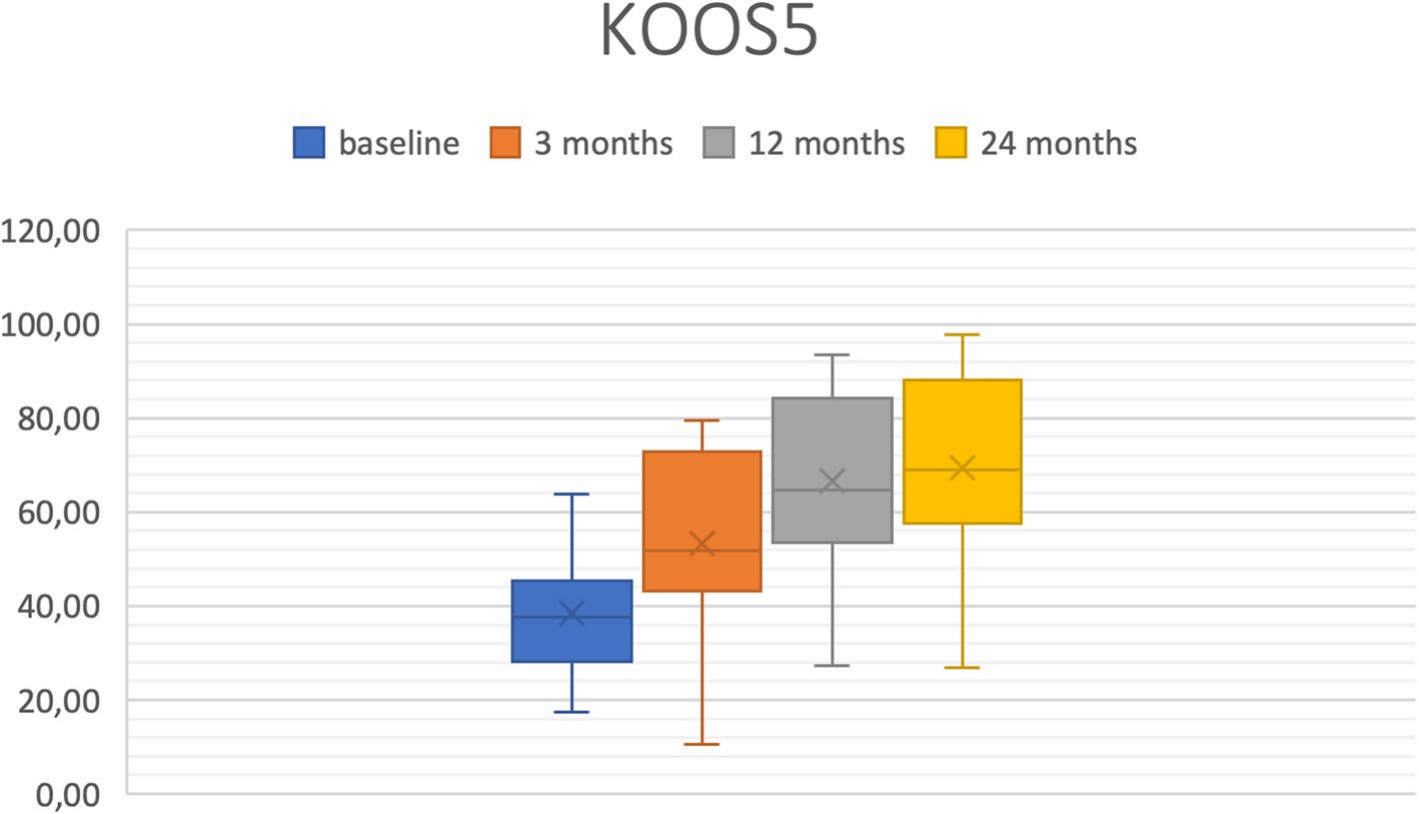
Boxplot: Aggregated Knee Injury and Osteoarthritis Outcome Score (KOOS5) *n* = 23 (*n* = 11 at 24 months)

The KOOS (KOOS5) showed substantial improvement in all five subdomains Tables 2 and 3, Fig. 9. Preoperatively, 75% of the patients presented with a KOOS5 below 45.3 (median = 37.7). After three months, 75% of the patients reported a KOOS5 above 43.3 (median = 51.7), after 12 months above 53.6 (median = 64.7) and after 24 months above 57.6 (median = 69.1).

**Table 3:** Statistical results for KOOS5 subdomains including mean, median, range, and standard deviation (SD), as well as first and third quartile (*n* = 23 after one year, *n* = 11 after two years). The number in the columns headings refers to the number of months postoperatively

	**Pain preOP**	**Pain3**	**Pain12**	**Pain24**	**Sym preOP**	**Sym3**	**Sym12**	**Sym24**	**ADL preOP**	**ADL3**	**ADL12**	**ADL24**	**Sport preOP**	**Sport3**	**Sport12**	**Sport24**	**QoL preOP**	**QoL3**	**QoL12**	**QoL24**
Min	16.7	13.9	30.6	33.3	21.4	10.7	28.6	32.1	26.5	11.8	38.2	36.8	0	0	5	5	0	0	12.5	12.5
Max	75	83.3	97.2	100	78.6	89.3	89.3	96.4	79.4	94.1	97.1	100	45	75	90	95	43.8	75	87.5	81.3
Mean	43.5	56.5	68.6	72.4	45.5	55.7	64.8	75	48.7	64.1	77	75.4	14.8	36.5	53.6	50	23.4	39.4	49.7	53.8
Standard deviation	15.5	20.5	21.6	21.1	16.8	20	17.8	17.3	16.2	20.6	17.7	20	13.9	23	24.4	29.6	11.5	20.7	25.1	24.3
1st quartile (exclusive)	33.3	50	55.6	61.1	28.6	39.3	57.1	71.4	36.8	50	64.7	60.3	0	20	37.5	23.8	12.5	25	25	34.4
Median	41.7	58.3	66.7	69.4	46.4	57.1	67.9	78.6	48.5	67.6	76.5	76.5	12.5	35	55	52.5	25	43.8	43.8	59.4
3rd quartile (exclusive)	50	75	91.7	97.2	60.7	71.4	82.1	82.1	57.4	80.9	95.6	94.1	26.3	55	75	80	31.3	56.3	75	76.6

*VAS* Visual Analogue Scale for Pain

*KOOS* Knee Injury and Osteoarthritis Outcome Score; KOOS5 represents the mean of all five subcategories

Quality of Life

*ADL* Activities of Daily Life

*Sym* Symptoms

### Failures and revisions

Up to now, there have been no cases of conversion to arthroplasty. However, after one year one patient (m, 64 years old) presented with persistent pain in the medial compartment without swelling and extension deficit of 5° in the knee. Arthroscopy revealed a degenerative tear of the medial meniscus as well as an anterior tibial osteophyte and scar tissue. After removing the osteophyte and part of the meniscus due to the degenerative nature of the tear, the patient presented again after 1.5 years with local swelling and pain near the origin of the medial collateral ligament (MCL). The pain prior to implantation of the patient-specific implants entirely disappeared. The second arthroscopy showed chronic inflammation of the MCL origin near the medial epicondyle. Debridement and intensive physiotherapeutic training led to significant improvement of pain.

## Discussion

This study showed major improvements of VAS for pain and KOOS in all five subdomains at three, 12, and 24 months after bicompartmental implantation of patient-specific implants. After three months, the third quartile was already below the first quartile of the preoperative examination, indicating good early results for 75% of the patients. Over the course of two years the median gradually decreased; however, the third quartile increased from 35 after 12 months to 48 after 24 months. While the KOOS5 presented with a slower increase, the median improved constantly as well. As for the slight increase of the third quartile of the VAS, more data will have to be gathered to identify outliers or possibly see a trend. The gradual improvement of the median in the current data, both for KOOS5 and VAS for pain, suggests that patients benefit from bicompartmental implantation of patient specific implants. The patients included in this study mostly matched the range of age for the treatment gap mentioned above. With 39 years, only one patient was younger, and five patients were older than 60 years. These patients previously underwent a failed biological repair and decided to receive two patient specific implants after discussing all possible treatment options. All patients were evaluated by the surgeon, however, and their respective biological age was considered to be in the range of 40 to 60 years. The range for age is not to be viewed as an absolute, but rather as a complement to the listed indications and contraindications.

Unicompartmental implantation of one patient specific implant has been evaluated by Holz et al., who published a prospective study of 75 patients two years after receiving treatment. The mean VAS improved from 63 preoperatively to 32 after 24 months, and the aggregated KOOS increased from 35 to 59. Two patients (2.5%) underwent revision, both due to atypical lesions and persisting pain [9].

A smaller study group of ten patients was prospectively analyzed by Al-Bayati et al. over a mean time of 75 months (SD 10). The results were comparable with a reduction of VAS for pain from 60 preoperatively to 26 after 24 months, and 24 after 75 months. The KOOS was not calculated as an aggregated KOOS score, but four of the five individual subcategories improved significantly [1].

In comparison to the results of one implant, our results for two implants in one knee do not appear to differ from the current literature. The VAS for pain even improved further, which might be due to the bifocal disease before surgery and a subsequently greater relief in pain.

Another comprehensive multicenter study published by Ryd et al. tracked 612 patients treated with 682 implants, with a follow-up of up to seven years. Fourteen patients (2.3%) had to be revised after seven years due to disease progression, incorrect implant positioning, and inadequate lesion coverage at the time of surgery [21].

The revision rate pointed out by Ryd et al. is comparable to the rate mentioned by Holz et al. (2.5%) and will have to be further confirmed for patients with two implants in one knee.

Moewis et al. investigated the knee kinematics 12 months after implantation of a patient-specific mini-metal implant. Data were collected using fluoroscopic analysis. The results for ten knees 12 months after surgery were compared to ten healthy knees. Further clinical data were collected via the VAS for pain, KOOS and Health EuroQol-5d (EQ 5d).

Physiological patterns were observed in medial pivot, lateral femoral rollback, and coupled axial external femoral rotation during flexion. Femoral rollback and axial external rotation were higher compared to the healthy knee, which Moewis et al. suggested could be caused by postoperative muscle weakness as differences persisted 12 months after surgery.

Focal resurfacing strategies for cartilage lesions have been discussed in the literature in the past years, including implants such as the HemiCAP, UniCap (Arthrosurface, USA) or BioPoly (Schwartz Biomedical, USA).

In 2011, Bollars et al. presented a consecutive series of 27 patients with a median age of 49 years who were treated with the femoral condyle HemiCAP [2]. The outcome was measured via KOOS, International Knee Documentation Comitee (IKDC), Hospital for Special Surgery Score (HSS), Western Ontario and McMaster Universities Osteoarthritis Index (WOMAC) and radiographs. The median follow up was 34 months (range 20-57). The KOOS showed good-to-excellent results after surgery in all domains, and the HSS improved significantly as well. The WOMAC-Score averaged 90.1 ± 9.3.

However, this study only included a small patient collective with a relatively short follow-up.

Pascual-Garrido compared 30 patients who were treated with a biological chondral repair to 32 patients who received a focal metallic resurfacing implant (Hemi-CAP) in a matched-pair analysis [18]. WOMAC, SF-12 and patient satisfaction were collected. After two years, 53% of the patients who underwent biological repair and 75% of patients treated with the Hemi-CAP-implant showed an improvement of 20% on WOMAC-Score. The SF-12 results only showed statistically significant improvement for the CAP-group. Excellent patient satisfaction was reported for 80% of the patients with biological repair and 91% with a metal implant. However, the results provided were only for short-term follow-up.

Laursen and Lind published long-term results over 7 years [12]. Sixty-one patients were examined. Thirty-six received a femoral Hemi-CAP-implant and 25 were treated with a trochlear implant (Hemi-Wave). Knee Society Score (KSS), pain scores and radiographic examination were determined over two years. Complications and reoperations were additionally collected within seven years after surgery.

Although the short-term results showed a promising improvement in KSS from 52 to 90, and in pain scores from 7.1 to 1.8, patients had to be revised in 23% of the cases in the seven-year follow-up. Considering this high revision rate, Laursen and Lind concluded that focal metal implants could only be considered a temporary treatment option for cartilage lesions.

Dhollander at al. came to a similar conclusion in a prospective study of 14 patients with a mean follow-up of 26.1 months [6]. All patients showed gradual clinical improvement, yet significant osteoarthritic changes were observed in the radiographic results even though implant positioning was considered adequate in all cases.

Dhollander et al. concluded that focal metal implants such as the Hemi-CAP could only be considered a salvage procedure in patients with previously failed biological chondral repair.

In their systematic review, published in 2018, Fuchs et al. noted that studies investigating the implant showed conflicting results [8]. Uncertainty remained concerning the progression of osteoarthritis and around one-fifth of all patients had to undergo arthroplasty after four years.

Partial or full knee arthroplasty are both considered the appropriate treatment for patients with corresponding cartilage lesions as an expression of end-stage osteoarthritis [4, 16, 20]. However, the results are worse in younger patients [11, 17]. Meehan et al. stated that patients younger than 50 years had a significantly higher risk of revision surgery [14]. Up to now, there are no studies comparing the outcome of arthroplasty with patient specific mini-metal implants.

As for now, focal cartilage lesions remain a challenge in terms of treatment. Salvage options have been proposed but showed high revision rates. Patient-specific implants may offer an improved outcome, but thus far have only been evaluated for covering one focal cartilage lesion. This study presents the first short-term results for patients that suffer from two femoral cartilage lesions in one knee. In the past, after failed biological repair, this oftentimes resulted in full knee arthroplasty. Providing an alternative option for middle-aged patients is crucial to avoid complications and revisions that come with early total joint replacement.

These first data on bicompartmental implantation of patient specific implants can, as for now, only be considered an alternative option for a rare indication.

### Limitations

This is a non-randomized study without a control group. It only evaluates the results over a comparatively short period of time. Additionally, with 23 patients (11 after two years), the number of patients is relatively low. Therefore, evaluation of the revision rate is non-representative, and this study could be biased in overestimating positive results. Furthermore, disease progression has only been monitored through patient-reported outcome measures without objective radiological imaging.

To conclude, this study can only be seen as a pilot study. A larger study lot must be conducted to examine patients over a longer period of time to provide reliable results.

## Conclusions

This pilot study presents promising results for bicompartmental implantation of patient-specific implants in middle-aged patients. In the future, use of patient specific implants in two compartments might delay a full arthroplasty to avoid subsequent revision surgery. To provide reliable results however, a bigger study lot must be investigated over a longer period of time.

### Supplementary Information

**Additional file 1.**

## Data Availability

All data supporting the findings of this study are available within the paper and its Supplementary Information.

## References

[1] Al-Bayati M, Martinez-Carranza N, Roberts D, Högström M, Stålman A (2022). Good subjective outcome and low risk of revision surgery with a novel customized metal implant for focal femoral chondral lesions at a follow-up after a minimum of 5 years. Arch Orthop Trauma Surg.

[2] Bollars P, Bosquet M, Vandekerckhove B, Hardeman F, Bellemans J (2012). Prosthetic inlay resurfacing for the treatment of focal, full thickness cartilage defects of the femoral condyle: a bridge between biologics and conventional arthroplasty. Knee Surg Sports Traumatol Arthrosc.

[3] Brittberg M, Gomoll AH, Canseco JA, Far J, Lind M, Hui J (2016). Cartilage repair in the degenerative ageing knee. Acta Orthop.

[4] Campi S, Tibrewal S, Cuthbert R, Tibrewal SB (2018). Unicompartmental knee replacement - Current perspectives. J Clin Orthop Trauma.

[5] Chen C, Li R (2019). Cementless versus cemented total knee arthroplasty in young patients: a meta-analysis of randomized controlled trials. J Orthop Surg.

[6] Dhollander AAM, Almqvist KF, Moens K, Vandekerckhove P-J, Verdonk R, Verdonk P, Victor J (2015). The use of a prosthetic inlay resurfacing as a salvage procedure for a failed cartilage repair. Knee Surg Sports Traumatol Arthrosc.

[7] Egloff C, Hirschmann MT, Moret C, Henle P, Ellenrieder M, Tischer T (2021). Total knee arthroplasty in the young patient-an update. Orthopade.

[8] Fuchs A, Eberbach H, Izadpanah K, Bode G, Südkamp NP, Feucht MJ (2018). Focal metallic inlay resurfacing prosthesis for the treatment of localized cartilage defects of the femoral condyles: a systematic review of clinical studies. Knee Surg Sports Traumatol Arthrosc.

[9] Holz J, Spalding T, Boutefnouchet T, Emans P, Eriksson K, Brittberg M, Konradsen L, Kösters C, Verdonk P, Högström M, Lind M (2021). Patient-specific metal implants for focal chondral and osteochondral lesions in the knee; excellent clinical results at 2 years. Knee Surg Sports Traumatol Arthrosc.

[10] Jeuken RM, van Hugten PPW, Roth AK, Timur UT, Boymans TAEJ, van Rhijn LW, Bugbee WD, Emans PJ (2021). A Systematic Review of Focal Cartilage Defect Treatments in Middle-Aged Versus Younger Patients. Orthop J Sports Med.

[11] Klit J, Jacobsen S, Rosenlund S, Sonne-Holm S, Troelsen A (2014). Total Knee Arthroplasty in Younger Patients Evaluated by Alternative Outcome Measures. J Arthroplasty.

[12] Laursen JO, Lind M (2017). Treatment of full-thickness femoral cartilage lesions using condyle resurfacing prosthesis. Knee Surg Sports Traumatol Arthrosc.

[13] Makris EA, Gomoll AH, Malizos KN, Hu JC, Athanasiou KA (2015). Repair and tissue engineering techniques for articular cartilage. Nat Rev Rheumatol.

[14] Meehan JP, Danielsen B, Kim SH, Jamali AA, White RH (2014). Younger age is associated with a higher risk of early periprosthetic joint infection and aseptic mechanical failure after total knee arthroplasty. J Bone Joint Surg Am.

[15] Murphy MP, Koepke LS, Lopez MT, Tong X, Ambrosi TH, Gulati GS, Marecic O, Wang Y, Ransom RC, Hoover MY, Steininger H, Zhao L, Walkiewicz MP, Quarto N, Levi B, Wan DC, Weissman IL, Goodman SB, Yang F, Longaker MT, Chan CKF (2020). Articular cartilage regeneration by activated skeletal stem cells. Nat Med.

[16] Pandit H, Gulati A, Jenkins C, Barker K, Price AJ, Dodd C, a. F, Murray DW, (2011) Unicompartmental knee replacement for patients with partial thickness cartilage loss in the affected compartment. The Knee 18(3):168–171 10.1016/j.knee.2010.05.003 20627734

[17] Parvizi J, Nunley RM, Berend KR, Lombardi AV, Ruh EL, Clohisy JC, Hamilton WG, Della Valle CJ, Barrack RL (2014). High Level of Residual Symptoms in Young Patients After Total Knee Arthroplasty. Clin Orthop.

[18] Pascual-Garrido C, Daley E, Verma NN, Cole BJ (2017). A Comparison of the Outcomes for Cartilage Defects of the Knee Treated With Biologic Resurfacing Versus Focal Metallic Implants. Arthroscopy.

[19] Perdisa F, Bordini B, Salerno M, Traina F, Zaffagnini S, Filardo G (2023). Total Knee Arthroplasty (TKA): When Do the Risks of TKA Overcome the Benefits? Double Risk of Failure in Patients up to 65 Years Old. CARTILAGE.

[20] Price A, Beard D, Thienpont E (2013). Uncertainties surrounding the choice of surgical treatment for “bone on bone” medial compartment osteoarthritis of the knee. The Knee.

[21] Ryd L, Flodström K, Manley MT (2020). Patient-Specific Implants for Focal Cartilage Lesions in The Knee: Implant Survivorship Analysis up to Seven Years Post-Implantation. Surg Technol Int.

[22] Wodzig MHH, Peters MJM, Emanuel KS, Van Hugten PPW, Wijnen W, Jutten LM, Boymans TA, Loeffen DV, Emans PJ (2022). Minced Autologous Chondral Fragments with Fibrin Glue as a Simple Promising One-Step Cartilage Repair Procedure: A Clinical and MRI Study at 12-Month Follow-Up. Cartilage.

